# Who Should Lead Academia Today? Rethinking Leadership Across Career Stages

**DOI:** 10.1002/ca.70110

**Published:** 2026-03-11

**Authors:** Joe Iwanaga, William Swartz, E. George Salter, R. Shane Tubbs

**Affiliations:** ^1^ Department of Neurosurgery, Clinical Neuroscience Research Center Tulane University School of Medicine New Orleans Louisiana USA; ^2^ Department of Neurology, Clinical Neuroscience Research Center Tulane University School of Medicine New Orleans Louisiana USA; ^3^ Department of Structural & Cellular Biology Tulane University School of Medicine New Orleans Louisiana USA; ^4^ Department of Neurosurgery and Ochsner Neuroscience Institute Ochsner Health System New Orleans Louisiana USA; ^5^ Dental and Oral Medical Center Kurume University School of Medicine Kurume Fukuoka Japan; ^6^ Division of Gross and Clinical Anatomy, Department of Anatomy Kurume University School of Medicine Kurume Fukuoka Japan; ^7^ Department of Anatomy and Cell Biology Louisiana State University School of Medicine New Orleans Louisiana USA; ^8^ Department of Cell Biology University of Alabama at Birmingham School of Medicine Birmingham Alabama USA; ^9^ Department of Anatomical Sciences St. George's University St. George's Grenada; ^10^ Department of Surgery Tulane University School of Medicine New Orleans Louisiana USA; ^11^ University of Queensland Brisbane Australia

**Keywords:** academia, accountability, anatomy, emerita, emeritus, leadership, retired, retirement

## Abstract

Leadership in academic institutions and professional societies plays a critical role in shaping the future of scholarship, governance, and educational equity. However, a persistent trend, particularly in long‐established organizations, reveals that retired faculty, such as professors emeriti, often fill executive leadership roles. While emeriti may continue to offer valuable mentorship and institutional memory, their appointment to decision‐making positions raises significant structural and ethical concerns. This commentary critiques the reliance on retired academics for active leadership, highlighting key risks including diminished accountability, generational disconnection, ethical incongruence, and leadership bottlenecks that impede the advancement of early‐ and mid‐career scholars. Drawing on governance literature and demographic data, the article calls for structural reforms that promote active, inclusive, and forward‐looking leadership models. Practical recommendations include revising governance bylaws, establishing advisory roles for emeriti, and fostering intergenerational partnerships to ensure sustainable academic leadership. The future of academia cannot be led solely by the past. It must be shared by those actively engaged in its present with the assistance of those who have experience in such roles before retirement.

## 1 Introduction

A troubling pattern persists in many academic disciplines, particularly within long‐established societies and federations: professors emeriti frequently hold leadership positions, such as president or board chair. Although these individuals are often respected for their past accomplishments, they are, by definition, retired from full‐time academic engagement. Despite no longer contributing to day‐to‐day research, teaching, or mentorship, they are placed in roles that shape policy and strategic vision. While many emeriti continue to provide valuable mentorship and insight, entrusting them with executive leadership roles raises important structural and ethical concerns for academic organizations navigating rapid global, technological, and generational shifts.

## 2 Why Retired Leadership Presents Structural Risks

This leadership model, though historically rooted, introduces several risks:

1. Reduced Accountability: Active academic leaders are subject to institutional oversight mechanisms, including performance reviews, student feedback, and research output expectations. Retired professors, removed from these evaluative systems, operate outside accountability structures. Governance literature emphasizes that legitimacy is grounded in responsibility and transparency (Cornforth 2003; Bovens 2007).
2. Generational and Contextual Disconnect: The academic landscape has changed dramatically over the past two decades. Emeriti leaders may lack direct experience with contemporary challenges such as precarious employment, competitive funding environments, digital scholarship, and interdisciplinary expectations. Studies show that younger scholars face heightened pressures that older models of academic leadership may not fully address (Ehrenberg 2012; Laursen and Austin 2020). In a survey of 20 scientific societies based in the US and UK, Bankston found that including early career researchers (ECRs) in the governance of scientific societies brings benefits to both the researchers and the organizations (Bankston et al. 2020). The study emphasized that giving ECRs a voice in decision‐making may also encourage other academic societies to offer them greater leadership opportunities.
3. Ethical Incongruence: Ethical leadership demands future‐oriented thinking and active responsibility. Emeriti leaders, while symbolically powerful, may no longer bear the consequences of institutional decisions. Brown noted that authentic ethical leadership links authority to responsibility (Brown et al. 2005).
4. Bottleneck to Leadership Development: Occupying senior roles without succession planning obstructs opportunities for early‐ and mid‐career scholars. This impedes innovation and limits diversity of leadership perspectives (Kezar and Eckel 2008; Gigliotti 2022). According to the 2022 ACE American College President Study, over 55% of college presidents were above the age of 60, while only 5% were under 50, highlighting a generational imbalance that hampers leadership renewal (American Council on Education 2022). Recent commentary in biomedical academia argues that the traditional hierarchical “pyramid” model of academic advancement is no longer sustainable. Rathmell and Shevde (2025) propose inverting this pyramid to position early‐career investigators at the forefront of leadership and innovation, supported—not overshadowed—by senior faculty. Their call reflects growing concern that career progression has become excessively prolonged, crowding senior ranks and limiting upward mobility. Such structural stagnation mirrors the leadership bottleneck observed in many academic societies.

To be clear, emeriti should be valued as mentors, advisors, and institutional memory holders and some will not have any of the risks as outlined above. The critique here is not against emeriti as individuals but against outdated structures that misuse honorary roles as substitutes for active leadership. They must be evaluated on their desire to strive for the health of the organization by working with the younger members.

## 3 Toward More Responsive and Inclusive Governance

1. Promote Internal Reform: Societies and institutions should revise their bylaws to reserve executive roles for currently active faculty. Including early‐ and mid‐career scholars on boards, committees, and task forces ensures that decisions reflect contemporary challenges and solutions.
2. Adopt Hybrid Advisory Models: Instead of eliminating emeriti from leadership structures, consider advisory councils that guide without executive authority. This leverages emeriti expertise while maintaining contemporary governance.
3. Support New Organizational Models: New scholarly communities may emerge to test alternative governance structures when entrenched leadership resists reform. These entities can prioritize transparency, inclusivity, and responsiveness by assembling interdisciplinary and intergenerational leadership.
4. Encourage Scholarly Dialogue: Academic publications, symposia, and podcasts can surface these debates. Platforms such as the *Chronicle of Higher Education* and *Inside Higher Ed* increasingly explore generational turnover, equity in leadership, and the future of professional societies (Bourdieu 1988; Brint 2005).
5. Foster Intergenerational Partnerships: True reform emerges from collaboration. Senior faculty still active in the field should mentor the next generation of leaders. Intergenerational governance models promote sustainability, knowledge transfer, and legitimacy (Bozeman and Boardman 2014).

## 4 Conclusion

Tradition should inform, but not hinder, progress. Leadership must be an active, ethically engaged, and forward‐looking responsibility, not a ceremonial reward for past service. Institutions that prioritize symbolic leadership over functional efficacy risk becoming disconnected from the needs of today's scholars. Emeriti mentors are important as institutional memory holders, and reforming governance structures to reflect active academic life is not betrayal but stewardship. In a time of global transformation, this shift is necessary and overdue.

## Disclosure

The first author is in their 40s, and the last is in their 50s. Both are active professors. All other co‐authors are professors emeriti.

## Data Availability

Data sharing not applicable to this article as no datasets were generated or analyzed during the current study.
